# Hypoxia induces rapid, STAT3 and ROS dependent, mitochondrial translocation of RelA(p65) and IκBα

**DOI:** 10.1042/BSR20192101

**Published:** 2019-09-16

**Authors:** Iglika G. Ivanova, Neil D. Perkins

**Affiliations:** Faculty of Medical Sciences, Institute for Cell and Molecular Biosciences (ICaMB), Newcastle University, Newcastle Upon Tyne, U.K.

**Keywords:** hypoxia, mitochondria, nuclear factor kappaB, reactive oxygen species

## Abstract

The nuclear factor-κB (NF-κB) family of transcription factors can directly or indirectly regulate many important areas of biology, including immunity, inflammation and cell survival. One intriguing aspect of NF-κB crosstalk with other cell signalling pathways is its regulation of mitochondrial biology, including biogenesis, metabolism and apoptosis. In addition to regulating the expression of mitochondrial genes encoded in the nucleus, NF-κB signalling components are also found within mitochondria themselves and associated with mitochondrial DNA. However, complete biochemical analysis of mitochondrial and sub-mitochondrial localisation of all NF-κB subunits has not been undertaken. Here, we show that only the RelA NF-κB subunit and its inhibitor IκBα reside within mitochondria, whilst p50 is found in the endoplasmic reticulum (ER). Fractionation of mitochondria revealed that only RelA was found in the mitoplast, the location of the mtDNA. We demonstrate that hypoxia leads to a very rapid but transient accumulation of RelA and IκBα in mitochondria. This effect required reactive oxygen species (ROS) but was not dependent on the hypoxia sensing transcription factor subunit HIF1α or intracellular Ca^2+^ release. We also observed rapid mitochondrial localisation of transcription factor STAT3 following hypoxia. Inhibition of STAT3 blocked RelA and IκBα mitochondrial localisation revealing a previously unknown aspect of crosstalk between these key cellular regulators.

## Introduction

The nuclear factor-κB (NF-κB) family of transcription factors regulate the expression of a vast array of genes in response to extra- and intracellular stimuli by binding to κB DNA responsive elements within their promoters [1]. An important regulator of immunity, inflammation, development, cell division and survival, NF-κB also participates in many additional processes via crosstalk with other signalling pathways [2]. Aberrant NF-κB activation is therefore associated with many diseases, including cancer, with tumour cells often exhibiting constitutively active NF-κB [3].

Mammalian NF-κB consists of five subunits, RelA (p65), RelB, c-Rel, p100/p52 and p105/p50, all of which contain the REL Homology Domain (RHD) that mediates DNA binding and dimer formation [4,5]. The C-termini of RelA, RelB and c-Rel also contain transactivation domains, essential for controlling the expression of target genes [6,7]. NF-κB subunits form homo or heterodimers, held in the cytoplasm in an inactivated state through interactions masking their nuclear localisation sequences. RelA and c-Rel are inactivated by the ankyrin repeat containing Inhibitor of NF-κB (IκB) proteins such as IκBα [8]. The NF-κB subunit precursors p100 and p105 have their own C-terminal ankyrin repeat domains and can also function as inhibitors [9]. Activation of NF-κB therefore requires the removal of these inhibitors, either via degradation of the IκB proteins or processing of p100 and p105 to the shorter forms p52 and p50, respectively [10–12]. IκBs are phosphorylated and targeted for proteasomal degradation via the IκB kinase (IKK) complex, consisting of IKKα, IKKβ and NEMO [13]. This allows the nuclear localisation of the active NF-κB complexes, followed by target gene promoter binding and transcriptional regulation. It is now emerging that apart from their functions in NF-κB signalling, components of this pathway also play other independent roles [14,15].

Mitochondria are double membrane organelles containing their own genetic material encoded on circular DNA molecules (mtDNA) that are essential for energy, metabolite and reactive oxygen species (ROS) production, as well as calcium storage and apoptosis [16,17]. Mitochondrial dysfunction can lead to many diseases and is a contributing factor to ageing and cancer development. NF-κB signalling can directly regulate at least three aspects of mitochondrial biology: biogenesis by controlling the levels of dynamin-like protein Opa1 required for mitochondrial fusion [18]; metabolism by regulating the expression of a number of mitochondrial genes encoded in the nucleus as well as the mtDNA [19–22]; and apoptosis [19,21–24]. In most cases, mitochondrial changes induced by NF-κB culminate in cell survival. Changes in mitochondrial metabolism through NF-κB activity could also contribute towards its tumour promotor function via suppression of p53 signalling [19,23].

RelA, p50 and IκBα have all been reported to reside within the mitochondria of cancer cell lines as well as in tissues [20–27], suggesting that some NF-κB effects on mitochondria might be direct. Mitochondrial RelA was found to associate with the D-loop region of mtDNA and alter cytochrome b (CYTB) expression but this was still observed in a DNA binding mutant form of RelA, suggesting it is likely to be an indirect interaction [20,21]. Additionally, analysis of publically available ENCODE chromatin immunoprecipitation (ChIP)-seq datasets of nuclear transcription factor occupancy on mtDNA also suggests that NF-κB does not associate directly with the mitochondrial genome [28]. Therefore, it is likely that mitochondrial NF-κB does not have a classical transcriptional role, but rather, alters gene expression of mtDNA encoded genes as a co-factor or through alternative mechanisms.

RelA can interact with the mitochondrial chaperone mortalin, in a manner antagonised by the p53 tumour suppressor, leading to increased O_2_ consumption and ATP production [23]. Mitochondrial RelA levels also increase during apoptosis driven by ANT1 overexpression and LPS stimulation in cell lines, as well as in rat retina cells after exposure to very bright light [22,26]. Fas-induced apoptosis, on the other hand, reduced mitochondrial RelA and IκBα levels [25]. In all these cases, RelA was required for cell survival, indicating that mitochondrial RelA may play a role in preventing apoptosis. However, IκBα has been reported to be mostly associated with the outer mitochondrial membrane (OMM) with only a small fraction residing inside mitochondria [25,27]. Activation of NF-κB signalling by TNFα or Fas decreases mitochondrial IκBα and plays an important role in preventing apoptosis, independently of its function during NF-κB signalling [20,25]. OMM-associated IκBα mediates the interaction of VDAC and HKII during apoptosis and is necessary for survival [27]. These anti-apoptotic functions of mitochondrial IκBα contribute to tumour survival after doxorubicin treatment [27]. Although a mitochondrial form of p50 has been reported to have DNA binding activity, no functional data are currently available regarding a mitochondrial role for this NF-κB subunit [20,21].

Given these important functions of RelA and IκBα in mitochondrial metabolism and apoptosis, we decided to perform a detailed analysis of both the presence and sub-mitochondrial localisation of all NF-κB subunits. We discovered that only RelA and IκBα were consistently found inside mitochondria, whilst p50 resided in the ER. RelA mitochondrial localisation depended on the OMM translocase TOM40 and the mitochondrial chaperone mortalin. Additionally, we identified hypoxia as a novel stimulus causing a transient increase in mitochondrial RelA and IκBα, independently of the main hypoxia transcription factor subunit HIF1α and calcium signalling. Instead, reactive oxygen species (ROS) production drives RelA, IκBα as well as STAT3 mitochondrial localisation. We also report that localisation of RelA and IκBα to hypoxic mitochondria is dependent on STAT3 signalling, revealing a new and unexpected aspect of crosstalk between these pathways.

## Materials and methods

### Cell culture

Human Embryonic Kidney 293 (HEK293); breast cancer MCF7 and MDA-MB-231; and osteosarcoma U2OS cell lines were maintained in DMEM supplemented with 10% FBS (Thermo Fisher), 2 mM L-glutamine (SLS) and 50 U Penicillin/Streptomycin (SLS). Prostate cancer PC3 cells were cultured in RPMI with 25 mM HEPES supplemented with 10% FBS (Thermo Fisher) and 2 mM L-glutamine (SLS), whilst primary normal human juvenile dermal fibroblasts (Promocell) were maintained in Fibroblast growth media supplemented with 2% supplement mix (Promocell) and 1% Penicillin/Streptomycin/Fungizone Solution (Promocell).

### Treatments

In hypoxia experiments, cells were incubated for the indicated times at 1% O_2_ in an *in vivo *400 hypoxia work station (Ruskin). DMOG (Sigma); Thaspsigargin (Enzo); TPCA1 (Stratech) and STATTIC (Sigma) were dissolved in DMSO. N-acetyl-L-cysteine (SLS) was diluted in water and adjusted to pH 7.5. Cells were treated accordingly at concentrations and times indicated in figure legends.

### Mitochondria isolation by differential centrifugation

Cells were homogenised with a 7 ml Dounce homogenizer in homogenisation buffer (0.6 M D-mannitol, 5 mM Tris, pH 7.6; 1 mM EGTA, 0.1% BSA, 1µg/ml Leupeptin, Pepstatin A and Aprotenin and 1 mM PMSF) and centrifuged at 400 × ***g*** for 10 min at 4°C to remove cell debris. Cytoplasmic and crude mitochondrial fractions were obtained by centrifugation of the supernatant at 11,000 × ***g*** for 10 min at 4°C. Protein concentration was measured by Bradford (BioRad) and the mitochondrial pellets and the cytosolic fraction were treated with 10 ng Proteinase K per µg of protein for 30 min on ice. The Proteinase K treated mitochondria were washed in homogenisation buffer and the concentrations of cytosolic and mitochondrial protein were measured by Bradford assay (Biorad), to allow equal loading for analysis by SDS/PAGE and immunoblotting.

### Purification of mitochondria-associated ER

Crude mitochondria were prepared by differential centrifugation and laid on a 15%/17.5%/20% OptiPrep Density Gradient Medium (Sigma). The mitochondria and ER fractions were separated at 100,000 × ***g*** with an Optima L-100 XP ultracentrifuge (Beckman Coulter) equipped with a SW40Ti rotor for 2 h at 4°C. The purified ER was harvested from the 20% layer and half was treated with 10 ng Proteinase K per µg of protein for 30 min on ice. Equal amounts of protein were further analysed by immunoblotting.

### Mitochondrial sub-fractionation

Crude mitochondria were prepared by differential centrifugation, incubated on a tube rotator with 0.2U DNAse per mg of mitochondria for 15 min at room temperature and sub-fractionated by swelling with 1 mM EDTA/10mM Tris, pH 7.4 on a tube rotator at 4°C. The remaining mitoplast containing only the inner mitochondrial membrane and the enclosed matrix was additionally treated with 5 ng proteinase K/µg of protein for 30 min on ice. Equal amount of protein was further analysed by immunoblotting.

### siRNA knock down

U2OS cells were seeded at a density of 400,000 cells/plate in 10 cm^2^ dishes and transfected in OptiMem (ThermoFisher) with 5 nM siRNA pools or single siRNAs with 5 µl INTERFERin (Polyplus Transfection) transfection reagent the following day. After 48 h, the cells were split into 15 cm^2^ dishes and transfected again with 7.5 nM siRNA pools with 7.5 µl INTERFERin transfection reagent in OptiMem. The cells were harvested and mitochondria isolated 48 h later by differential centrifugation. Protein levels in the mitochondrial and cytosolic fractions were assessed by Bradford assay (BioRad) and mitochondria were further treated with 10 ng Proteinase K per µg of protein for 30 min on ice. The siRNA used were as follows: Scramble 5′ CAGUCGCGUUUGCGACUGG; RelA siRNA 1 5′ GCUGAUGUGCACCGACAAG; RelA siRNA 2 5′ GCCCUAUCCCUUUACGUCA; IκBα 5′ ACACUCAGCUCAUAAUA; TOM40 siRNA 1 ACUGAACAACUGGUUGG; TOM40 siRNA 2 CCCUCUGUAUGAAAUAG; TOM40 siRNA 3 GAAGAUGACUAUUUCAU; TIM50 siRNA 1 5′ AAUGUUGGCUUCUAACUAA; TIM50 siRNA 2 5′ GGUCUGUACUGUCCAGUAC; Mortalin 5′ CGAGUUGAAGCAGUUAAUA.

### Immunoblotting

Protein quantity was measured with Bradford reagent (BioRad) and prepared in SDS loading dye. For detecting HIF1α, a sample of the cells was harvested in 8 M Urea, sonicated and the protein quantified with the BCA assay (ThermoFisher). Between 5 and 10 µg of protein per well was loaded on SDS/PAGE (BioRad), separated and transferred with the Trans Blot Turbo semi dry system (BioRad) to PVDF (Millipore) membranes. The following antibodies were used for the present study at a dilution 1:1000: RelA C-terminal (sc-372, SantaCruz); RelA N-terminal (#4764, Cell Signalling); IκBα (#9242, Cell Signalling); p105/p50 (#3035, Cell Signalling); p100/p52 (#4882, Cell Signalling); RelB (#4954, Cell Signalling); c-Rel (#4727, Cell Signalling); α-tubulin (#2144, Cell Signalling); VDAC (#4866, Cell Signalling); protein disulphide isomerase (PDI) (sc-20132, Santa Cruz); TOM40 (sc-11414, Cell Signalling); AIF (sc-13116, Cell Signalling); TIM50 (SBS0666, Source BioSciense); Mortalin (MA3-028; Thermo Fisher); calnexin (#2433, Cell Signalling); HIF1α (610958, BD Transduction Laboratories); STAT3 (#9139, Cell Signalling).

### Immunofluorescence of saponin permeabilised cells

U2OS cells were grown on cover slips and exposed to hypoxia as indicated in figure legends. The cells were permeabilised with Solution A (7.23 mM K_2_EGTA, 2.77 mM CaK_2_EGTA, 60 mM MES, 20 mM taurine, 3 mM K_2_HPO_4_, 0.5 mM DTT, 81 mM CH_3_KO_3_S, pH 7.1) supplemented with 2.88 mg/ml MgATP and 70 µg/ml saponin for 10 min on ice according to [29]. After three washes with Solution A containing MgATP, cells were fixed in 3.7% Formaldehyde for 10 min, permeabilised with 1% Triton-X/0.05% Tween 20 for 15 min, washed in PBS and blocked for 30 min in 10% BSA in PBS/0.05% Tween 20. Slides were stained for 1 h with a 1:200 dilution of RelA sc-372 antibody (Santa Cruz) in 1% BSA/PBS/0.05% Tween20, washed in PBS and stained in the dark with a 1:500 dilution of goat anti-rabbit IgG DyLight 550 (Thermo Fisher) in 1% BSA/PBS/0.05% Tween20 for 30 min. After three more washes with PBS, slides were mounted with ProLong Gold mounting media containing DAPI (Invitrogen) and imaged with a Nikon A1R inverted confocal microscope.

## Results

### Only RelA from the NF-κB family of transcription factors, as well as IκBα reside inside mitochondria

Several studies have demonstrated that members of the NF-κB signalling pathway can be present within mitochondrial protein extracts, including RelA, p50, IκBα, IKKα, IKKβ and IKKγ, where they are thought to control apoptosis and mitochondrial metabolism [20,21,23,26,27]. However, a full investigation of the mitochondrial presence of all NF-κB subunits has not been undertaken. Moreover, mitochondria are closely associated with the ER [30] and some protocols are not able to distinguish between localisation in different compartments of the mitochondria or potential ER contamination.

**Figure 1:**
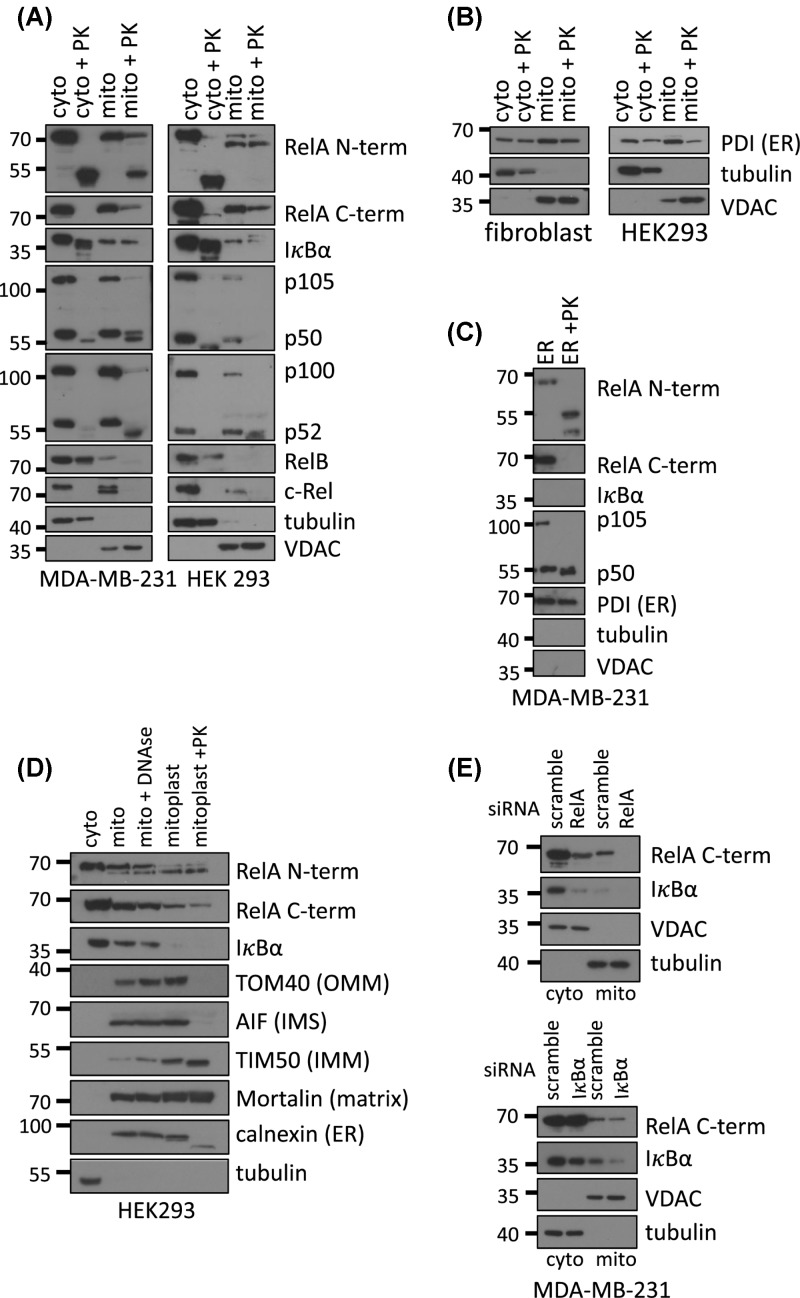
RelA and IκBα reside in mitochondria Protein levels from the indicated sub-cellular fractions were analysed by immunoblotting. Mitochondrial and cytosolic extracts were prepared from MDA-MB-231 (**A**) and HEK293 (**A, B**) cells by differential centrifugation. Both fractions were treated with 10 ng/µg of protein with proteinase K for 30 min on ice. (**C**) The ER from MDA-MB-231 cells was separated from crude mitochondrial extracts on a 15%/17.5%/20% OptiPrep gradient. Pure ER was collected in the 20% fraction and treated with 10 ng/µg of protein with proteinase K for 30 min on ice. (**D**) Mitochondria from HEK293 cells were treated with 0.2 U DNAse per mg of mitochondria for 15 min at room temperature and sub-fractionated by swelling with 10 mM Tris, pH 7.4. The remaining mitoplast containing only the inner mitochondrial membrane and the enclosed matrix was additionally treated with 5 ng/µg of protein with proteinase K from 30 min on ice. (**E**) RelA and I**κ**B**α** were knocked down with siRNA in MDA-MB-231 cells and proteinase K-treated mitochondria were prepared as in A. Data are representative of a minimum of two independent experiments.

To investigate the mitochondrial localisation of NF-κB subunits, crude mitochondria were purified by differential centrifugation of cellular homogenates containing intact organelles from p53 wild-type HEK293 and p53 mutant MDA-MB-231 cells to account for the known inhibitory effects of p53 expression on mitochondrial NF-κB [19,23]. Mitochondria isolated by this method, confirmed by the presence of the mitochondrial outer membrane protein VDAC, were free of cytosolic contamination, as shown by the lack of tubulin (Figure 1A). Examination of all NF-κB subunits in MDA-MB-321 and HEK293 cells corroborated previous findings that RelA, IκBα and p50 are associated with mitochondria [20,21,23,26,27]. Additionally, p105, p100/p52 and c-Rel but not RelB were also associated with the organelle (Figure 1A). However, only RelA and IκBα in both cell lines and p50 in MDA-MB-231 cells were resistant to proteinase K digestion, utilised to remove proteins unshielded from the mitochondrial membranes. This suggested that whilst p50 might only be associated with mitochondria in cells with mutant p53, RelA and IκBα can localise to mitochondria independently of p53 status. (Figure 1A). In HEK293 cell, we also noticed that with the N-terminal RelA antibody we detected a lower mobility band present only in the mitochondria that was also proteinase K resistant. However, RelA siRNA knock down indicated that this band was non-specific (data not shown). This data indicates that even though p105, p100/p52 and c-Rel might exist in proximity to mitochondria, they are unlikely to reside inside the organelle.

Further analysis demonstrated that mitochondria isolated through differential centrifugation also contained a proteinase K resistant fraction of the ER, confirmed by the presence of the ER lumen chaperone PDI (Figure 1B). Although this observation is not unusual, given the physical and functional interactions between these organelles [30], it was essential to discount the possibility of ER localisation of RelA, IκBα or p50. Crude ER/mitochondria preparations from MDA-MB-231 cells were, therefore, further separated on a Percol gradient to obtain a purified ER fraction. Proteinase K treatment of this fraction revealed that although RelA and IκBα were degraded, p50 remained intact in the ER, suggesting that its apparent localisation in mitochondrial extracts in MDA-MB-231 cells (Figure 1A) might originate from contaminating ER (Figure 1C). We therefore only focused on RelA and IκBα in subsequent experiments.

Next, mitochondria were subjected to further fractionation by swelling and removal of the OMM to obtain the mitoplast, the matrix enveloped by the inner mitochondrial membrane (IMM) that contains the mitochondrial genome. Interestingly, RelA localised partially to the proteinase K-treated mitoplast whilst IκBα did not (Figure 1D).

To confirm antibody specificity, we performed RelA or IκBα siRNA knockdown in highly transfectable MDA-MB-231 cells and observed reduced signals for both proteins in mitochondria (Figure 1E). RelA knockdown also reduced the levels of cytosolic IκBα, although this may reflect a reduction in IκBα transcription, which is under the control of RelA [31]. The loss of mitochondrial RelA following IκBα siRNA knockdown was unexpected and could be due to IκBα acting as a chaperone to RelA.

### TOM40 and mortalin are required for RelA mitochondrial localisation

Most mitochondrial proteins are nuclear encoded and need to be imported into mitochondria post-translation. The classical pathway for mitochondrial import is through the translocase of the outer membrane (TOM) followed by the translocase of the inner membrane (TIM) [32]. These are large multiprotein complexes forming pores through the mitochondrial membranes to deliver proteins into the intermembrane space and matrix by using ATP and the mitochondrial membrane potential.

**Figure 2:**
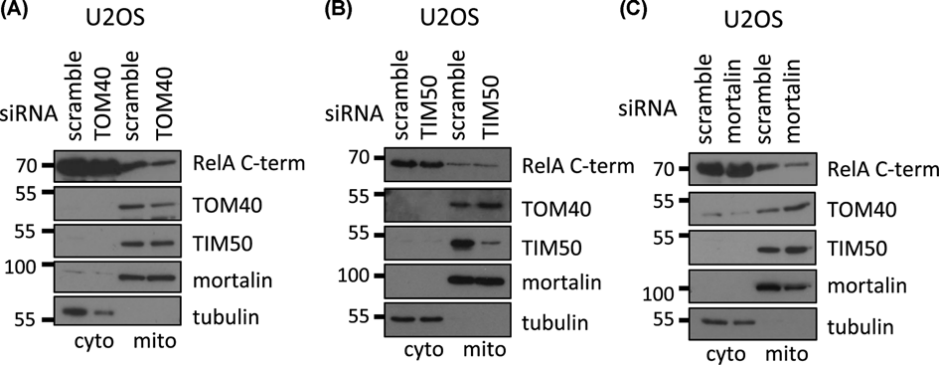
TOM40 and mortalin are required for the mitochondrial import of RelA Mitochondrial and cytosolic extracts were prepared by differential centrifugation from U2OS cells, where TOM40 (**A**), TIM50 (**B**) or mortalin (**C**) were knocked down with siRNA. The mitochondrial fractions were treated with 10 ng/µg of protein with proteinase K for 30 min on ice. Protein levels in each fraction were analysed by immunoblotting as indicated. Data are representative of a minimum of two independent experiments.

To identify which translocase is involved in RelA localisation into mitochondria we used siRNA to knockdown subunits of both translocases in highly transfectable U2OS cells and obtained proteinase K treated-mitochondria by differential centrifugation. RelA mitochondrial import depended on TOM40, a component of the TOM complex that forms a barrel traversing the outer mitochondrial membrane (Figure 2A). However, TIM50, one of three integral membrane proteins of the TIM translocase, was not involved in intrinsic RelA mitochondrial localisation (Figure 2B), although this experiment did not examine localisation to the mitoplast.

Our previous findings identified the matrix protein mortalin as a RelA interactor, crucial for its mitochondrial import [23]. Indeed, mortalin knockdown reduced the mitochondrial levels of RelA, confirming that it is needed for RelA mitochondrial localisation (Figure 2C).

### Hypoxia triggers rapid and transient mitochondrial translocation of RelA and IκBα

**Figure 3:**
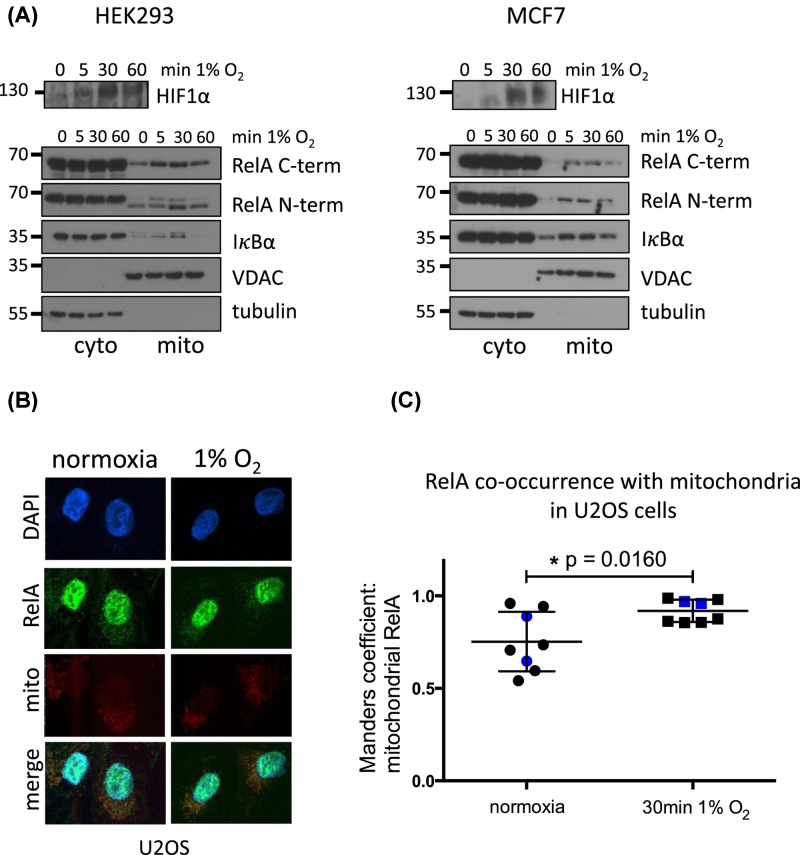
Hypoxia induces a transient RelA and IκBα translocation into mitochondria (**A**) Cytosolic and mitochondrial extracts were prepared by differential centrifugation from HEK293 and MCF7 cells cultured for the indicated times at 1% O_2_. Protein levels in each fraction were assessed by immunoblotting as indicated. Data are representative of a minimum of two independent experiments. (**B**) U2OS cells were grown on cover slips, mitochondria were stained with MitoTracker DeepRed FM (Invitrogen), exposed to 1% O_2_ for 30 min, cytosolic RelA was removed by permeabilisation with 70 µg/ml saponin on ice under hypoxia and fixed with formaldehyde. Organelle-associated RelA was stained with sc-372 primary antibody (Santa Cruz) and goat anti-rabbit IgG DyLight 550 (Thermo Fisher). Confocal Z stack images were obtained with a Nikon A1R inverted microscope. (**C**) A total of eight normoxic and eight hypoxic cells from three images each were analysed with the Huygens Colocalization Analyser [64]. Regions of interest that include the mitochondria but exclude the nucleus in individual cells were measured and the Manders’ Overlap Coefficient was calculated for each cell. The values depicted in blue on the graph correspond to the cells shown in (B). The error bars represent standard deviation and statistical analysis was performed in Prism Software with a two-tailed, unpaired *t-*test.

We were curious about the functional significance of RelA and IκBα localisation to mitochondria, specifically in the context of cellular signalling known to regulate their activity. A number of nuclear transcription factors have been found to translocate to mitochondria during hypoxia [33–35]. Therefore, we asked if RelA and IκBα could also do so by exposing cells to 1% O_2_ and preparing proteinase K-treated mitochondria. Indeed, we observed that both RelA and IκBα translocated to mitochondria within minutes of cells being subjected to hypoxia, with levels decreasing after 1 h (Figure 3A). This was further confirmed by immunofluorescence (Figure 3B,C). Due to the high cytosolic presence of RelA, normoxic and hypoxic U2OS cells were first permeabilised with saponin and the cytosol was washed out, leaving only organelle associated RelA. Analysis of confocal images revealed that RelA colocalised with mitochondria significantly more during hypoxia (Figure 3B,C). It is, therefore, likely that RelA and IκBα are implicated in the early stages of hypoxia, rather than during later stages of adaptation via transcription.

### Elevated mitochondrial RelA and IκBα levels during hypoxia are ROS dependent

Hypoxia activates NF-κB via IKK, independently of signalling through the oxygen sensing hypoxia-inducible transcription factor HIF1α, although in hypoxia activation does not involve degradation of IκBα [36,37]. However, lack of oxygen in cells and tissues leads to several additional rapid physiological changes, occurring within minutes of exposure to hypoxia, such as a burst of mitochondrial ROS and superoxide levels [38,39], an increase in intracellular Ca^2+^ levels [40,41] and accumulation of HIF1α [42]. ROS and superoxide generation during hypoxia mainly originates from the Q cycle in complex III of the mitochondrial electron transport chain [43,44] and is accumulated in the first 30 min after which levels return to basal level [39,45].

To determine if any of these events are involved in mitochondrial RelA and IκBα localisation during hypoxia, we first treated MDA-MB-231 cells with the hypoxia mimetic DMOG, which inhibits the PHD enzymes responsible for HIF1α hydroxylation and proteolytic degradation, leading to accumulation of HIF1α without lowering O_2_ levels.

**Figure 4:**
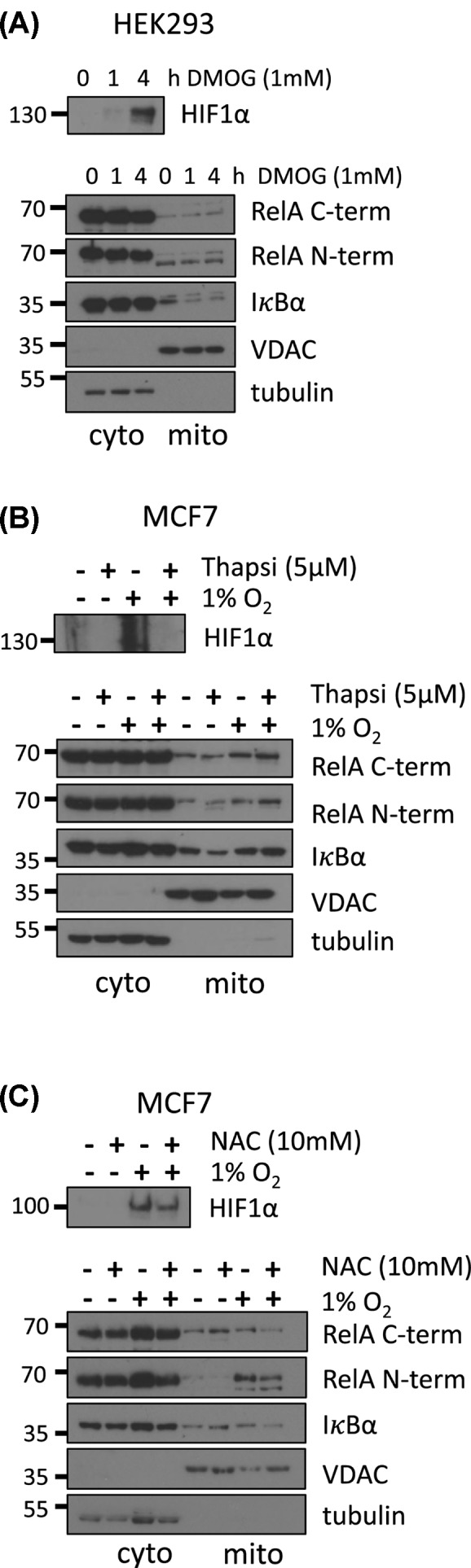
Mitochondrial translocation of RelA and IκBα during hypoxia is dependent on ROS (**A**) Cytosolic and mitochondrial extracts were prepared by differential centrifugation from MDA-MB-231 cells treated with 1 mM of the hypoxia mimetic DMOG for 1 and 4 h (**B**). MCF7 cells were treated with 5 µM Thapsigargin (**C**) or 10 mM NAC for 1h (**D**) and then exposed to 1% O_2_ for 30 min before mitochondria and cytosolic fractions were isolated. All mitochondrial fractions were treated with 10 ng/µg of protein with proteinase K for 30 min on ice. Protein levels in each fraction were assessed by immunoblotting as indicated. Data are representative of a minimum of two independent experiments.

DMOG treatment did not alter mitochondrial RelA or IκBα levels (Figure 4A). This finding was reinforced by using a chemical inhibitor of HIF1α translation, thapsigargin that blocks the production of HIF1α protein by preventing the association of the RNA binding protein YB1 with the 5′UTR of HIF1α mRNA [46]. Thapsigargin is also known as a sarco/endoplasmic reticulum Ca^2+−^ATPase (SERCA) inhibitor that depletes the ER calcium stores and results in accumulation of intracellular Ca^2+^, another event associated with early hypoxia. Cells treated with thapsigargin showed no changes to RelA and IκBα mitochondrial translocation either with or without hypoxia exposure (Figure 4B). Taken together these findings suggest that HIF1α hydroxylation and accumulation, as well as Ca^2+^ release do not appear to trigger RelA and IκBα mitochondrial translocation (Figure 4A,B). Instead, cells treated with the antioxidant N-acetyl-L-cysteine (NAC) prevented the mitochondrial translocation of RelA and IκBα during hypoxia, suggesting mitochondrial ROS production is required for mitochondrial RelA and IκBα accumulation (Figure 4C).

### STAT3 signalling is required for RelA and IκBα mitochondrial accumulation during hypoxia

**Figure 5:**
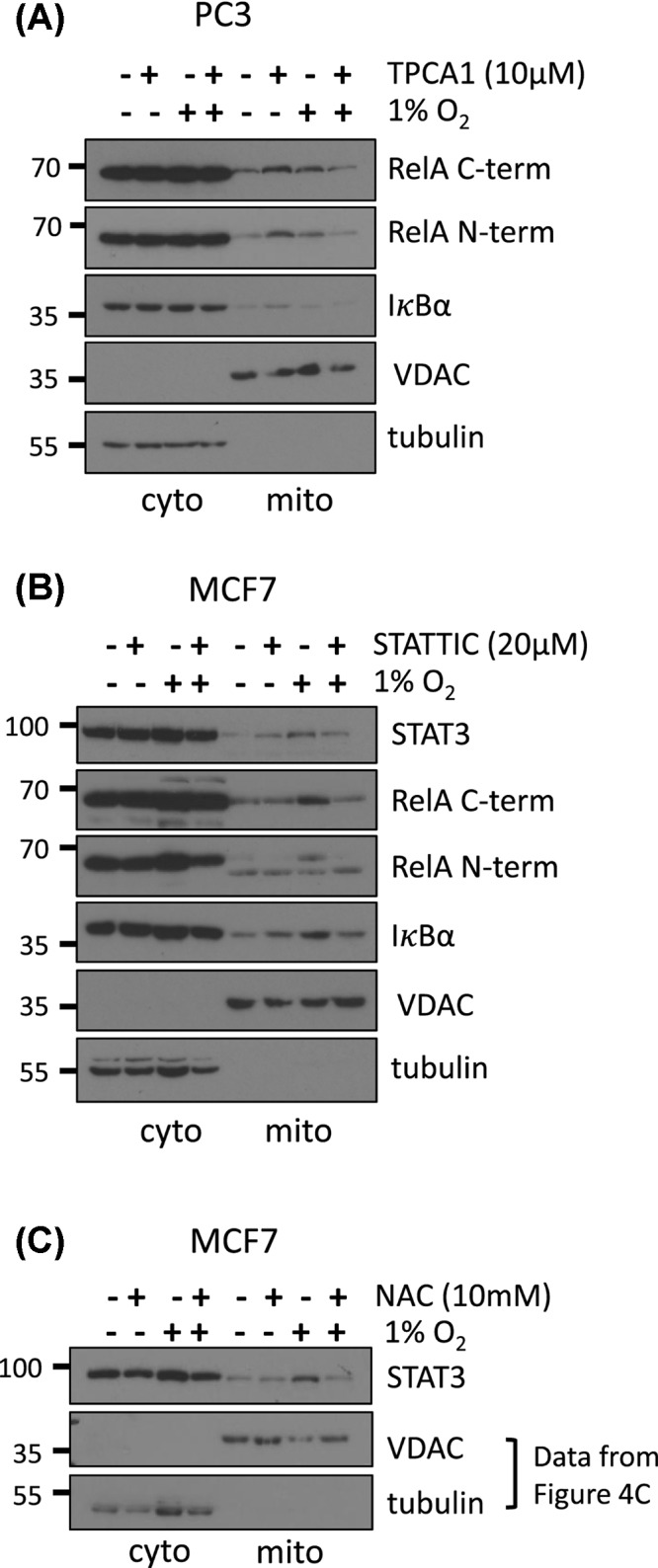
Inhibition of NF-κB or STAT3 signalling impairs hypoxia dependent RelA and IκBα mitochondrial translocation PC3 cells were treated with 10 µM TPCA1 (**A**) or MCF7 cells were treated with 20 µM STATTIC (**B**) then exposed to 1% O_2_ for 30 min. Cytosolic and mitochondrial extracts were prepared by differential centrifugation and mitochondrial fractions were incubated with 10 ng/µg of protein with proteinase K for 30 min on ice. (**C**) Hypoxia- and NAC-treated extracts from Figure 3D were analysed by immunoblotting for STAT3 levels. Data are representative of a minimum of two independent experiments.

Hypoxia can activate NF-κB within 5 min of exposure via IKKβ [36]. These kinetics are strikingly similar to our observation of RelA and IκBα mitochondrial translocation within 5 min of hypoxia (Figure 3A). To establish if IKK signalling was needed for mitochondrial translocation of RelA and IκBα, we utilised the ATP competitive IKKβ inhibitor TPCA1. TPCA1 is also known to inhibit STAT3, a nuclear transcription factor that translocates to mitochondria during hypoxia, by binding to its SH2 dimerisation domain and preventing phosphorylation of adjacent sites required for activity [33,47]. To address this off target effect, we obtained proteinase K-treated mitochondria from the STAT3 negative cell line PC3 [48]. TPCA1 reduced mitochondrial RelA and IκBα levels in PC3 cells during hypoxia, indicating that active NF-κB signalling is required for their mitochondrial accumulation (Figure 5A). We also observed an increase of RelA and IκBα in normoxia following TPCA1 treatment, which could be due to off target effects.

To further delineate the pathway required for RelA and IκBα mitochondrial accumulation, we utilised STATTIC, an irreversible inhibitor of STAT3. Similarly to TPCA1, STATTIC also inhibits the SH2 dimerisation domain and adjacent phosphorylation sites of STAT3, resulting in lack of DNA binding and loss of Tyr^705^ and Ser^727^ phosphorylation [49,50]. Interestingly, STAT3 positive MCF7 cells treated with this inhibitor accumulated less STAT3, RelA and IκBα into mitochondria during hypoxia, suggesting that active STAT3 can also regulate the mitochondrial association of these factors (Figure 5B). Moreover, NAC also reduced mitochondrial STAT3 levels in cells undergoing hypoxia, implicating ROS in mitochondrial STAT3 signalling (Figure 5C). This is consistent with the role of mitochondria targeted STAT3 in decreasing ROS production by the electron transport chain of ischemic mouse hearts [33]. It is therefore likely that hypoxia-associated ROS production acts as a signal to rapidly recruit STAT3, RelA and IκBα to mitochondria.

## Discussion

In the present study, we established that of the NF-κB subunits, only RelA together with its inhibitor IκBα, are consistently found inside the mitochondria of unstimulated cancer cells (Figure 1A,C). Moreover, we investigated the mitochondrial import route for RelA (Figure 2). As described previously, we confirmed that the mitochondrial chaperon mortalin interacts with RelA and is required for RelA mitochondrial localisation (Figure 2C) [23]. Mortalin aids the import of a subset of mitochondrial proteins by binding to them in the cytosol and facilitating their docking to the OMM translocase [51,52]. The OMM component TOM40 was also required for mitochondrial RelA localisation (Figures 1D and 2A). A proportion of RelA was also found in the mitoplast (Figure 1D). This biochemical evidence is consistent with previous ChIP data demonstrating that RelA can alter mitochondrial metabolism by associating with mtDNA indirectly [23]. In contrast, IκBα was not found in the mitoplast, suggesting it functions differently to RelA, perhaps in preventing apoptosis as previously shown [27].

Intriguingly, we identified hypoxia as a novel stimulus resulting in rapid and transient accumulation of RelA and IκBα into mitochondria, independently of HIF1α accumulation and Ca^2+^ release (Figures 3 and 4). STAT3 also accumulated rapidly in mitochondria following hypoxia (Figure 5B,C). STAT3 mitochondrial import is well defined. Similarly to RelA, STAT3 also uses a chaperone for import as well as for integration into complex I within the IMM [53]. Furthermore, Ser^727^ phosphorylation and K87 acetylation of STAT3 are crucial for mitochondrial import [53,54].

Inhibition of NF-κB and STAT3 signalling, with TPCA1 and STATTIC, respectively, reduced mitochondrial RelA and IκBα during hypoxia (Figure 5). STATTIC and TPCA1 can both prevent Ser^727^ phosphorylation of STAT3 [47,49,50], thereby, inhibiting STAT3 mitochondrial localisation simply by reducing mitochondrial import. STATTIC has also been found to reduce NF-κB activation and DNA binding, presumably due to crosstalk with STAT3 signalling [50,55]. A direct inhibitory effect of STATTIC on NF-κB, however, cannot be excluded.

STATTIC treatment also leads to increased mitochondrial ROS production and opening of the mitochondrial permeability transition pore (MPTP), a conductance channel at the IMM that leads to mitochondrial swelling, rupture of the OMM and apoptosis [56,57]. We therefore can also not rule out that reduction of RelA and IκBα mitochondrial levels after STATTIC treatment might be due to loss of the integrity of the mitochondrial membranes and consequent leakage of mitochondrial proteins.

Mitochondrial STAT3 regulates mitochondrial complex I and complex II activity [58,59]; controls pyruvate to acetyl-CoA conversion, ATP production, fatty acid synthesis and the mitochondrial potential following insulin stimulation [54]. It also suppresses mitochondrial ROS production, preventing MPTP opening and apoptosis [56–58,60,61]. It is therefore likely that during hypoxia STAT3 might play a similar role in suppressing ROS production and apoptosis (Figure 5C).

Mitochondrial ROS generated during hypoxia can activate the redox sensing tyrosine kinase c-SRC which in turn can stimulate both NF-κB and STAT3 through tyrosine phosphorylation on IκBα and STAT3 [37,62]. Interestingly, the kinetics of rapid ROS accumulation observed in the literature [39,45] mirror the kinetics of RelA and IκBα accumulation following exposure to hypoxia (Figure 3A). Consistent with ROS being implicated in activation of these pathways, we observed that the antioxidant NAC reduced mitochondrial RelA, IκBα and STAT3 during hypoxia (Figures 4C and 5C).

Similar to STAT3, IκBα has also been implicated in preventing apoptosis [27]. Inhibition of NF-kB signalling following hypoxia in *ex vivo* cultured rat ventricular myocytes leads to mitochondrial potential loss, MPTP opening and caspase 9 activation, suggestion that the anti-apoptotic role of NF-κB is mediated through the mitochondria [63]. Taken together our data and the available literature point to a scenario in which mitochondrial RelA, IκBα and STAT3 cooperatively participate in preventing apoptosis during the early stages of hypoxia.

## Abbreviations

ANT1: Adenine Nucelotide Transporter 1
ChlP: chromatin immunoprecipitation
c-Rel: REL Proto-Oncogene, NF-κB subunit
c-SRC: SRC Proto-Oncogene, Non-Receptor Tyrosine Kinase
DMOG: dimethyloxaloylglycine
DTT: Dithiothreitol
ER: endoplasmic reticulum
HEK293: human embryonic kidney 293
HEPES: (4-(2-hydroxyethyl)-1-piperazineethane sulfonic acid
HIF1α: Hypoxia Inducilbe Factor 1α
HKII: Hexokinase 2
IκB: inhibitor of NF-κB
IKK: IκB kinase
IMM: inner mitochondrial membrane
LPS: Lipopolysaccharide
MES: 2-(N-morpholino)ethanesulfonic acid
MPTP: mitochondrial permeability transition pore
mtDNA: mitochondrial DNA
NAC: N-acetyl-L-cysteine
NEMO: NF-κB Essential Modulator
NF-κB: nuclear factor-κB
OMM: outer mitochondrial membrane
PMSF: Phenylmethylsulfonyl fluoride
p65: RELA Proto-Oncogene, NF-κB subunit
RelA: RELA Proto-Oncogene, NF-κB subunit
RelB: RELA Proto-Oncogene, NF-κB subunit
ROS: reactive oxygen species
RPMI: Roswell Park Memorial Institute (RPMI) 1640 medium
SLS: Scientific Laboratory Supplies Ltd
STATTIC: Selective STAT3 inhibitor, 6-Nitrobenzo[b]thiophene 1,1 dioxide
STAT3: Signal Transducer and Activator of Transcription 3
TIM: translocase of the outer membrane
TOM: translocase of the outer membrane
TPCA1: 2-[(aminocarbonyl)amino]-5-(4-fluorophenyl)-3-thiophenecarboxamide
VDAC: Voltage Dependent Anion Channel
